# Hyde and Montgomery on the Skin

**Published:** 1904-11

**Authors:** 


HYDE AND MONTGOMERY ON THE SKIN. A Practical Treatise on Diseases of the Skin, for the use of students and practitioners. By James Nevins Hyde, M.D., Professor of Dermatology and Venereal Diseases, and Frank H. Montgomery, Associate Professor of Dermatology and Venereal Diseases in Rush Medical College, Chicago. Seventh and revised edition. In one octavo volume of 938 pages, with 107 engravings and 35 plates in colors and monochrome. Cloth, $4.50, *net*; leather, $5.50, *net*. Lea Brothers & Co., Philadelphia and New York, 1904.

This edition has been brought down to date as fully as seems warranted by the crystallization of recent advances. Some new diseases have been included and some old are classified. The Fray and Ainsen treatments are given due prominence and credit as useful and permanent additions to our curative methods. The illustrations of diseases are about as perfect as they can be made in view of the fact that many skin diseases can not be recognizably pictured. The principles of dermatology are not so perfectly laid down as seems desirable, but in general clearness and fullness the work is most admirable and deserves a standard place.

